# Transcriptome Analysis and Identification of Sesquiterpene Synthases in Liverwort *Jungermannia exsertifolia*

**DOI:** 10.3390/bioengineering10050569

**Published:** 2023-05-09

**Authors:** Xiaoguang Yan, Yukun Li, Weiguo Li, Dongmei Liang, Shengxin Nie, Ruiqi Chen, Jianjun Qiao, Mingzhang Wen, Qinggele Caiyin

**Affiliations:** 1 Department of Pharmaceutical Engineering, School of Chemical Engineering and Technology, Tianjin University, Tianjin 300072, China; 2 Key Laboratory of Systems Bioengineering (Ministry of Education), Tianjin University, Tianjin 300072, China; 3 SynBio Research Platform, Collaborative Innovation Center of Chemical Science and Engineering (Tianjin), Tianjin 300072, China; 4 Zhejiang Institute of Tianjin University, Shaoxing 312300, China

**Keywords:** *Jungermannia exsertifolia*, liverwort, microbial terpene synthase-like, transcriptome analysis

## Abstract

The liverwort *Jungermannia exsertifolia* is one of the oldest terrestrial plants and rich in structurally specific sesquiterpenes. There are several sesquiterpene synthases (STSs) with non-classical conserved motifs that have been discovered in recent studies on liverworts; these motifs are rich in aspartate and bind with cofactors. However, more detailed sequence information is needed to clarify the biochemical diversity of these atypical STSs. This study mined *J. exsertifolia* sesquiterpene synthases (JeSTSs) through transcriptome analysis using BGISEQ-500 sequencing technology. A total of 257,133 unigenes was obtained, and the average length was 933 bp. Among them, a total of 36 unigenes participated in the biosynthesis of sesquiterpenes. In addition, the in vitro enzymatic characterization and heterologous expression in *Saccharomyces cerevisiae* showed that JeSTS1 and JeSTS2 produced nerolidol as the major product, while JeSTS4 could produce bicyclogermacrene and viridiflorol, suggesting a specificity of *J. exsertifolia* sesquiterpene profiles. Furthermore, the identified JeSTSs had a phylogenetic relationship with a new branch of plant terpene synthases, the microbial terpene synthase-like (MTPSL) STSs. This work contributes to the understanding of the metabolic mechanism for MTPSL-STSs in *J. exsertifolia* and could provide an efficient alternative to microbial synthesis of these bioactive sesquiterpenes.

## 1. Introduction

**Figure 1:**
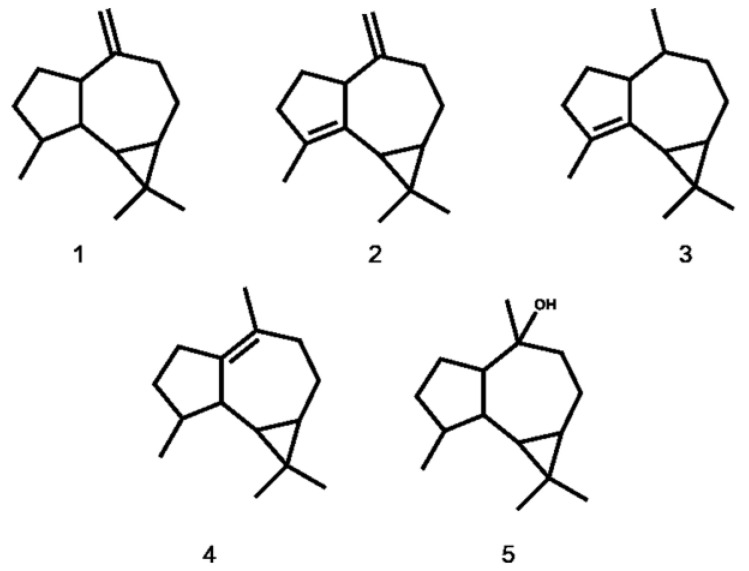
Aromadendrene-type sesquiterpenes of Jungermanniaceae liverworts. 1, aromadendrene; 2, aromadendra-4,10(14)-diene; 3, *α*-gurjunene; 4, viridiflorene; 5, viridiflorol.

Liverworts are considered to be primitive terrestrial plants with relatively simple morphologies but a high diversity of secondary metabolites [1,2]. These ubiquitous plants are rarely eaten by animals or infected by bacteria or fungi due to their unique bioactive compounds, especially sesquiterpenes, in their tissues [3]. Many antifungal and insecticidal compounds have been extracted from liverworts and their applications in medical treatment have a long history [4]. More interestingly, the terpene profiles of Jungermanniaceae have strong species specificity and are mainly comprised of aromadendrene-type sesquiterpenes (Figure 1) [2,5], such as viridiflorol isolated from Jungermanniaceae with biological activities of inhibiting bacterial growth and biofilm formation [6].

The formation of sesquiterpenes is catalyzed by the sesquiterpene synthases (STSs), most of which contain two critical aspartate-rich motifs, the DDXXD motif and the (N, D) DXX (S, T) XXXE motif, in the entrance of the enzyme cave [7]. These motifs form ion clusters with their cofactor Mg^2+^, and they have a coordination interaction with the pyrophosphate group of the substrate farnesyl diphosphate (FPP) to form a stable complex. The catalytic process is triggered by the removal of the pyrophosphate group from FPP, leading to the generation of the carbocation intermediate [8]. The active carbocation intermediate undergoes a series of cyclization reactions and rearrangement reactions to form carbocations with different sesquiterpene structures. Eventually, a series of sesquiterpene products is formed by deprotonation or capture of nucleophiles (usually H_2_O) [9].

Recently, microbial terpene synthase-like (MTPSL) proteins, a new branch of plant terpene synthases, were first identified in *Selaginella moellendorffii*. They are phylogenetically distant to the archetypical plant STSs due to their lower sequence similarity, the atypical aspartate-rich DDXXD motif for cofactor binding, and their protein structure composed of only an α-domain [10]. However, the identified MTPSL-STSs are limited to a few non-seed plant species, such as liverworts, hornworts, mosses, and lycophytes [11]. In addition, the biological function of MTPSL-STSs is still unknown. Further characterization of MTPSL-STSs from more plant species will give insight into the biological role of this new clade of plant terpene synthases.

*Jungermannia exsertifolia*, a special liverwort belonging to the Jungermanniaceae family, is distributed in a subtropical area in China. Jungermanniaceae species have oval oil bodies containing sesquiterpenes and their derivatives [4]. Some sesquiterpene oxides and phenolic compounds have been identified in *J. exsertifolia* exhibiting notable antioxidant, antibacterial activity, and ultraviolet light-absorbing function [12,13]. However, the *J. exsertifolia* sesquiterpene synthases (JeSTSs) used for the biosynthesis of these sesquiterpenes with special structures and the phylogenetic position of the JeSTSs are still unknown.

In recent years, sequencing technology has advanced by leaps and bounds. RNA sequencing has been used to research the transcriptome of model and non-model species [14]. Because of this, a vast number of STSs have been explored by analyzing the transcriptomes of various organisms, which has provided abundant data to support the research of the metabolic synthesis and biological function of terpenoids [15,16]. In addition, the abundance of transcript sequence reads is beneficial for exploring the biological responses to environmental stresses [17], identifying the evolutionary position of enzymes [18], analyzing the biosynthesis pathways for the bioactive secondary metabolites [19], and discovering the specific regulation of different biochemical processes [20]. To date, there has been no research on the transcriptome of *J. exsertifolia*. In this study, transcriptome analysis was performed using BGISEQ-500 sequencing technology. A total of 36 unigenes participating in the biosynthesis of sesquiterpenes was mined. Six putative JeSTSs were identified, three of which showed sesquiterpene catalytic activity in vitro and in *Saccharomyces cerevisiae*. Furthermore, the identified JeSTSs had a phylogenetic relationship with MTPSL-STSs. This work contributes to the understanding of the metabolic mechanisms of MTPSL-STSs in *J. exsertifolia* and could provide an efficient alternative to microbial synthesis of these bioactive sesquiterpenes.

## 2. Materials and Methods

### 2.1. Plant Materials and RNA Purification

*J. exsertifolia* was collected in August 2017 from Mount Cangshan, 25 degrees 40 min north latitude, 100 degrees 40 min east longitude, Yunnan Province, China. The plant tissues were frozen at −80 °C. An RNAprep pure Plant Kit was used to extract the total plant RNA. The specific operation was carried out according to the methods and the steps in the instructions, and the consumables used were sterilized after treatment with DEPC water. A NanoDrop 2000 (Thermo Fisher Scientific, Waltham, MA, USA) was used to measure the concentration of the RNA samples. The OD_260_/OD_280_ ratios of all the samples were between 1.8 and 2.1, which indicated that they could be used for library construction. An Agilent 2100 Bioanalyzer (Agilent Technologies, Santa Clara, CA, USA) was used to evaluate the RNA integrity number of each sample. Samples with an RNA integrity number >7.0 were considered to have no signs of degradation and were chosen for the following research.

### 2.2. Extraction and Analysis of Essential Oils from Jungermannia exsertifolia

The organic solvent extraction method [21] was used to extract the essential oils from *J. exsertifolia*. The samples were placed in a round-bottom flask, 300 mL methanol was added, and a heating jacket and condensation reflux device were connected. The extraction was carried out at 69 °C for 8 h. The extract was collected and placed in a rotary evaporator to concentrate the samples. The concentrated extract was washed several times with a small amount of n-hexane before being subjected to GC-MS analysis.

An Agilent 7890B-7000D GC-MS system equipped with an automatic sampler was used to analyze the sample components. The chromatographic column used for GC-MS was HP-5MS (30 m × 0.25 mm × 0.25 μm). The ion source was the electron ionization. The detector temperature was 200 °C, and the transmission line temperature was 230 °C. The split injection with an injection volume of 1 μL was performed at 280 °C, and the split ratio was 1:10. The heating program maintained the oven temperature for 2 min at 70 °C, then raised the temperature to 290 °C at 10 °C/min, and maintained it for 2 min. The mass spectrometry (MS) scanning range was 35–350 *m/z*. The carrier gas was helium with a flow rate of 1 mL/min. Qualitative analysis of the components was conducted based on comparing the relative retention indexes (RIs) and MS spectra with the spectra of the standard or spectra in the NIST17 library. The area normalization method was used to calculate the percentage of each component for quantitative analysis.

### 2.3. cDNA Library Construction and BGISEQ-500 Sequencing

The cDNA library was constructed following a previously described method [22]. There were two biological replicates (J1 and J2). A SMART^®^cDNA Library Construction Kit (TaKaRa Bio, Mountain View, CA, USA) was used to synthesize the cDNA. Then, a BGISEQ-500 system was used to sequence each cDNA library. The specific methods and steps were performed according to the instructions of Beijing Genomics Institute (BGI-Shenzhen, Shenzhen, Guangzhou, China).

### 2.4. De Novo Assembly and Functional Annotation Analysis of Unigenes

Before analyzing the transcriptome data, the low-quality reads were removed from the raw data. The low-quality reads included reads containing adaptors, reads containing bases with a mass value below 10 accounting for more than 20% of the total number of bases, and reads containing more than 5% of the unknown base N. The filtered reads were named “clean reads”. Next, Trinity (v2.0.6) [23] was used to assemble clean reads, and TGICL (v2.0.6) [24] was used to cluster and to make de-redundant the assembled transcripts in order to obtain unigenes.

After that, functional annotation of the unigenes was performed. Several genes in the same cluster were named after the CL with the number of the gene family due to their high similarity (general similarity >70%), while genes without clusters were named after the unigene. BLASTn and BLASTx were used to compare unigenes with nucleic acid databases and protein databases with an E-value threshold of 10^−5^. The nucleic acid database was the NCBI non-redundant nucleotide sequence (NT) database, and the protein databases were the NCBI non-redundant protein (NR) database, Kyoto Encyclopedia of Genes and Genomes protein (KEGG) database, SwissProt database, and euKaryotic Orthologous Group (KOG) database. In addition, Blast2GO [25] was used to generate the Gene Ontology (GO) annotations of the unigenes. Then, GO classification was conducted in order to show the distribution of gene functions [26]. Meanwhile, the InterPro annotations of the unigenes were obtained using InterProScan5 program.

### 2.5. Selection and Phylogenetic Analysis of Candidate JeSTSs

Candidate JeSTSs containing the conserved terpene synthase domains (Pfams: PF01397, PF03936, and PF06330) were obtained based on InterPro annotations. Putative JeSTS sequences were aligned using ClustalW. MEGA 6.0 evolutionary analysis software was used to construct a neighbor-joining phylogenetic tree [15]. The sequence information of the proteins participating in the phylogenetic analysis is shown in Table S1.

### 2.6. Functional Characterization of JeSTSs In Vitro

The candidate genes synthesized after codon optimization were amplified by PCR. The PCR products were subcloned into the pET28a vector to obtain the recombinant protein expression vector. The primers used are given in Table S2. After transforming the recombinant protein expression vector into *E. coli* BL21 (DE3), the correct single colonies were picked and inoculated into 10 mL Luria–Bertani (LB) medium comprising 50 μg/mL kanamycin, and cultured overnight at 37 °C. The preculture broth was inoculated into 200 mL of the same medium and cultured at 37 °C until the OD_600_ reached 0.8. The protein expression of candidate genes was induced after adding 0.5 mM isopropyl-1-thio-β-D-galactopyranoside (IPTG) into the culture medium. The induction temperature was 16 °C. After induction, the bacterial cells were collected by centrifugation, resuspended in phosphate buffered saline, and then lysed by an ultrasonic crusher until the bacterial solution became clear and bright. After ultrasonication, 20 μL of the total lysate, supernatant and precipitated resuspension, was boiled with 5 μL 5× supersample buffer for 10 min, and then the induction of the recombinant protein was identified by SDS-PAGE. The remaining supernatant of the lysate was purified using a His-bound NI-NTA affinity resin column (Genescript, Nanjing, China). The specific methods and steps were performed in accordance with the manufacturer’s protocol. The size of the purified protein was verified using SDS-PAGE.

The purified protein or the lysate containing the target protein was mixed with 50 mM Tris-HCl, 10 mM MgCl_2_, and 20 μM FPP (Sigma-Aldrich) to identify the protein function in vitro. The total mixture volume was 100 μL which was reacted at 30 °C for 1 h [27]. Then, 1 M EDTA and 4 M NaOH were added to terminate the reaction, and 200 μL n-hexane was added to extract the reaction products, which were detected using GC-MS.

### 2.7. Heterologous Expression of JeSTSs in Saccharomyces cerevisiae

The candidate genes were subcloned into the pESC-LEU vector. Then, the expression vector was transformed into the engineered *S. cerevisiae* strain Sc027 for heterologous expression. Sc027 is a strain that overexpresses mevalonic acid (MVA) pathway-related genes [28]. The primers used are shown in Table S2. The correct transformants were selected and inoculated into 10 mL leucine-deficient synthetic medium comprising 20 g/L glucose, 5 g/L (NH_4_)_2_SO_4_, 2 g/L amino acid mix lacking leucine, and 6.7 g/L yeast nitrogen base, and then cultured at 30 °C for 18 h. The preculture broth was transferred into 50 mL of the same medium in the appropriate proportion to make the initial OD_600_ of the culture broth equal to 0.05. The culture broth was cultured at 30 °C for 120 h. At 30 h, the expression of *GAL* promoter-controlled genes was induced after adding galactose to a final concentration of 10 g/L. Alcohol was added to a final concentration of 10 g/L as a supplementary carbon source for microbial growth at 36 h and 60 h of cultivation, respectively. After 120 h of cultivation, 5 mL hexane was added to extract the products, which were detected using GC-MS.

## 3. Results

### 3.1. Chemical Composition of Essential Oils from Jungermannia exsertifolia

The organic solvent extraction method was used to extract the essential oils from *J. exsertifolia*. Qualitative and quantitative analyses of its chemical composition was conducted using GC-MS. Table 1 shows that there were nine sesquiterpenes detected and the aromandendrene-type sesquiterpenes aromadendrene (23.5%) and viridiflorol (21.3%) were identified in high proportions.

### 3.2. RNA Sequencing and Transcriptome Assembly

After removing the low-quality reads, a total of 110.35 and 110.57 Mb of clean reads was obtained with a percentage of Q30 ≥ 80% (Table S3). Subsequently, a total of 162,624 and 118,139 unigenes was obtained, and the average lengths were 939 and 863 bp, respectively (Table 2). The raw reads are stored in the NCBI Sequence Read Archive (SRA) database with the SRA accession number PRJNA614598.

After de novo assembly, a total of 257,133 unigenes was obtained. The average length was 933 bp and N50 was 1921 bp (Table 2). There were 69,983 unigenes with a length longer than 1000 bp. The detailed results of the length distributions are shown in Figure S1.

**Table 2:** Quality metrics of unigenes.

Sample	Total Length	Mean Length	Total Number	GC (%)	N50 ^a^	N70 ^b^	N90 ^c^
J1	152,809,976	939	162,648	48.64	2004	982	320
J2	101,978,767	863	118,139	48.97	1685	838	309
All unigenes	239,995,006	933	257,133	48.72	1921	973	322

^a^ N50: N50 is used to evaluate the continuity of assembly, and a higher value indicates a better assembly effect. N50 is the length of the transcript that is added last, when the transcripts are sorted by length from largest to smallest, accumulating the length of transcripts sequentially to the length of the transcripts that are not less than 50% of the total length; ^b^ N70: similar to N50; ^c^ N90: similar to N50.

### 3.3. Functional Annotation of Transcriptome

After assembly, the gene functions of the unigenes were annotated based on the databases NT, NR, KEGG, SwissProt, KOG, GO, and InterPro. A total of 142,802 transcripts (55.54% of assembled transcripts) matched the sequences in these well-curated databases. Additionally, a total of 114,656 transcripts (44.59% of assembled transcripts) showed similarity to the sequences in the NR database (Table 3).

**Figure 2:**
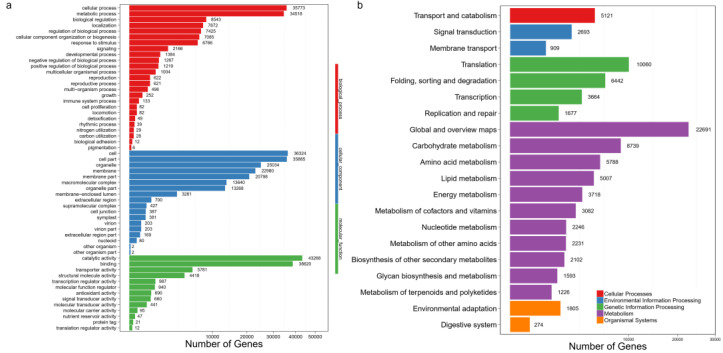
Functional distribution of GO (**a**) and KEGG (**b**) annotations.

As shown in Figure 2a, a total of 78,862 transcripts (30.67% of assembled transcripts) was categorized into 56 groups which were divided into the three main GO terms including “biological process”, “cellular component”, and “molecular function”. In “biological process”, the most representative GO category was cellular process, followed by metabolic process. There were 18 groups in “cellular component”, and a total of 36,324 and 35,885 unigenes was classified into cell and cell part, respectively. Under the 13 groups of “molecular function”, most unigenes were assigned to catalytic activity and binding groups.

The analysis of the KEGG annotations was also conducted to reveal the genes’ functions in metabolic pathways. From the *J. exsertifolia* transcriptome, a total of 88,847 unigenes had related annotations and were distributed into 20 KEGG groups. “Metabolism” pathways possessed the largest number of unigenes. In addition, a total of 1226 unigenes was assigned to the metabolism of terpenoids and polyketides. These gene functional annotations provide useful clues for mining the metabolic pathways of specific sesquiterpenes for *J. exsertifolia* (Figure 2b).

### 3.4. Sequence Information of Candidate JeSTSs

In order to explore JeSTSs of *J. exsertifolia*, the genes coding the enzyme containing terpene synthase conserved domains (Pfams: PF01397, PF03936 and PF06330) based on the InterPro annotations were considered as candidates. Six putative *JeSTSs* (Table S4) with ORFs from the *J. exsertifolia* transcriptome were subsequently identified. The coding sequences of the *JeSTSs* are stored in the NCBI GenBank database with the accession numbers MT277446 (*JeSTS1*), MT277447 (*JeSTS2*), MT277448 (*JeSTS3*), MT277449 (*JeSTS4*), MT277450 (*JeSTS5*), and MT277451 (*JeSTS6*).

**Figure 3:**
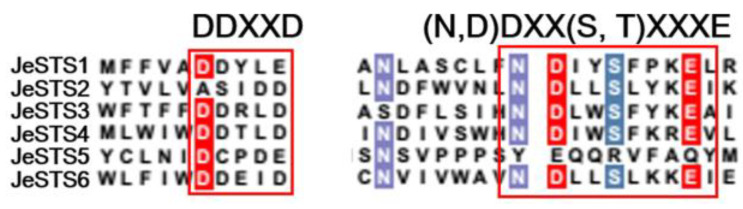
Sequence alignment of putative JeSTSs for *Jungermannia exsertifolia*. Cofactor-binding motifs are highlighted in red boxes.

**Figure 4:**
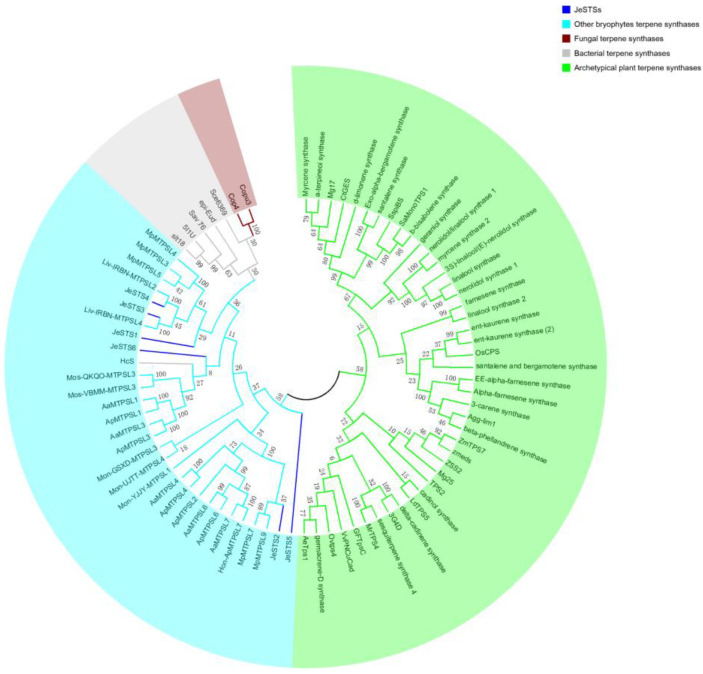
Phylogenetic analysis of putative JeSTSs for *Jungermannia exsertifolia*.

JeSTS1, JeSTS2, JeSTS3, JeSTS4, JeSTS5, and JeSTS6 consist of 397, 353, 450, 379, 387, 564, and 351 amino acids, respectively. Their predicted molecular weights are 46.2 kDa, 40.2 kDa, 51.5 kDa, 43.8 kDa, 63.1 kDa, and 39.6 kDa, respectively. The sequence alignment results of the JeSTSs indicated that the cofactor binding (N, D) DXX (S, T) XXXE motif is conserved in JeSTSs with the exception of JeSTS5. However, the conserved aspartate-rich DDXXD motif for cofactor binding of STSs was replaced by atypical aspartate-rich regions like DDXXE in JeSTS1 and XXXDD in JeSTS2 (Figure 3). The sequence similarity of JeSTSs is lower than the archetypical plant STSs and the highest sequence identity is 22.7% between JeSTS1 and JeSTS4. Phylogenetic analysis of the JeSTSs with some known bryophyte, bacterial, fungal, and vascular plant terpene synthases indicated that JeSTSs have a closer relationship with bryophyte STSs and microbial STSs than the archetypical plant STSs (Figure 4).

### 3.5. Functional Identification of JeSTSs In Vitro

**Figure 5:**
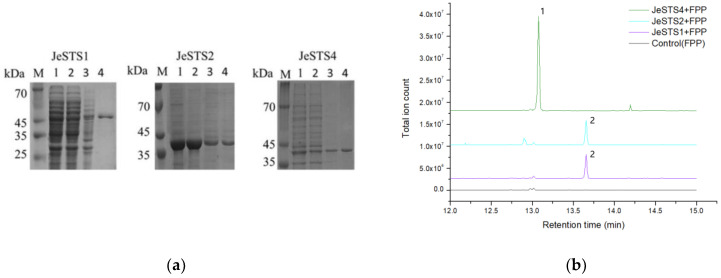
(**a**) SDS-PAGE analysis of JeSTSs. M, marker; 1, total protein; 2, supernatant; 3, precipitation; 4, purified protein. (**b**) GC-MS analysis of the reaction products from in vitro assay for JeSTSs. Peak 1 is bicyclogermacrene. Peak 2 is nerolidol.

After induction with IPTG at 16 °C, the recombinant purified proteins of JeSTS1, JeSTS2 and JeSTS4 were obtained (Figure 5). The other JeSTSs contained target bands after induction, but the target proteins were located within the inclusion body, resulting in the inability to obtain purified proteins. The purified protein or the lysate containing the target proteins was mixed with the terpene synthase substrate FPP to conduct in vitro reaction. The GC-MS results showed that recombinant proteins JeSTS1 and JeSTS2 could catalyze the formation of linear sesquiterpene nerolidol from FPP. Additionally, JeSTS4 could catalyze the formation of bicyclogemarene from FPP. However, no products were detected after the lysates of the other JeSTSs had reacted with the substrate in vitro, and their functions need to be further identified in vivo.

### 3.6. Heterologous Expression of JeSTSs in Saccharomyces cerevisiae

**Figure 6:**
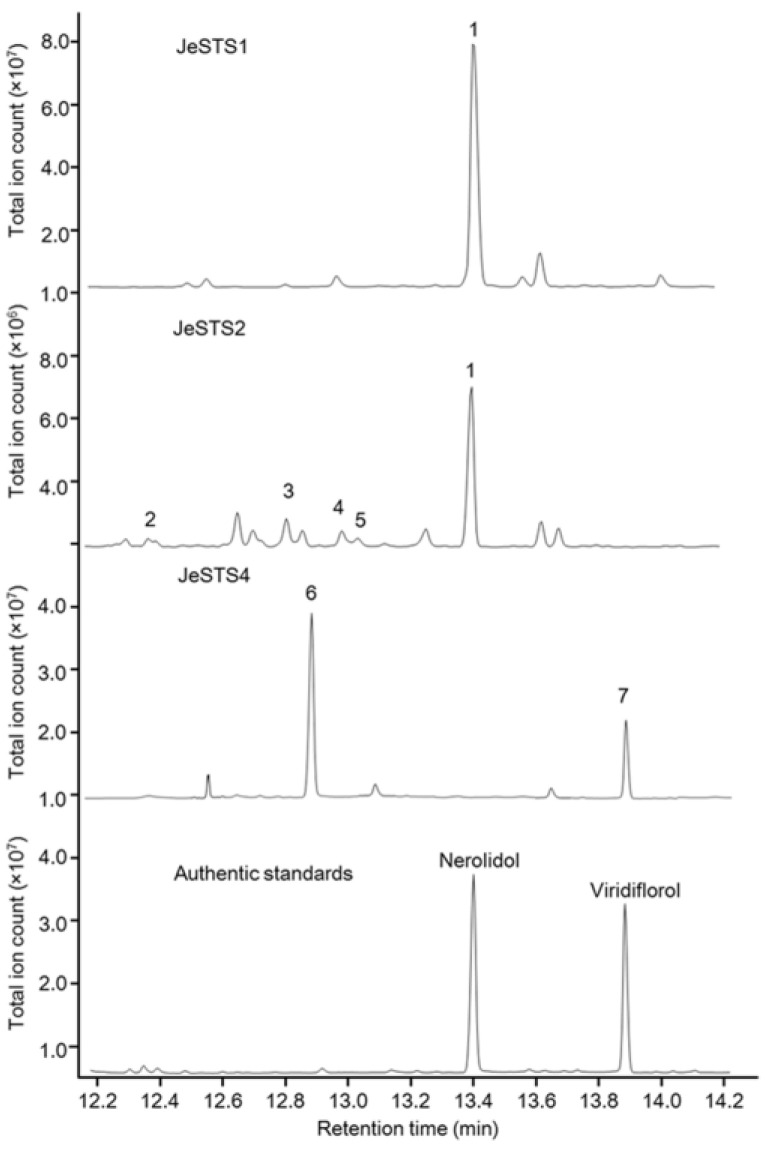
GC-MS analysis of products of JeSTSs in *Saccharomyces cerevisiae*. Peaks of the GC trace: 1, nerolidol; 2, *β*-funebrene; 3, *β*-chamigrene; 4, *β*-himachalene; 5, dauca-4(11), 8-diene; 6, bicyclogermacrene; 7, viridiflorol. The comparisons of the mass spectra of the peaks with the authentic standard mass spectra and the compound mass spectra stored in the NIST17 library are shown in Figure S2.

The candidate genes were subcloned into the pESC-LEU vector. Then, the expression vector was transformed into the engineered *S. cerevisiae* strain Sc027. The GC-MS detection results of the fermentation broth extract are shown in Figure 6. JeSTS1 produced nerolidol while JeSTS2 and JeSTS4 produced many sesquiterpene products. JeSTS2 catalyzed the conversion of FPP to nerolidol as the dominating product, and it also produced small amounts of *β*-funebrene, *β*-chamigrene, *β*-himachalene, and dauca-4(11), 8-diene. JeSTS4 mainly produced bicyclogermacrene and viridiflorol. However, JeSTS3, JeSTS5, and JeSTS6 did not form sesquiterpene products, which was consistent with the results of the in vitro functional identification, indicating that they had no sesquiterpene synthase activity.

## 4. Discussion

Liverworts are rich in secondary metabolites with complex structures and various biological activities [4,29]. Sesquiterpenes constitute the largest class of secondary metabolites in liverwort. Most of these sesquiterpenes are volatile with strong odor and can protect liverworts from insects and parasites [30,31]. However, the application of these sesquiterpenes is limited by their extraction from morphologically small plant materials. At present, the mining of STSs has attracted extensive attention, and the new STSs have greatly improved the application field of natural products [32]. *J. exsertifolia* accumulates specific sesquiterpenes, such as aromadendrene and viridiflorol in its essential oils (Table 1) which have biological activities [33]. Therefore, this study attempted to conduct an analysis of the transcriptome of *J. exsertifolia* in order to find more STSs responsible for the biosynthesis of bioactive sesquiterpenes. After de novo assembly, a total of 257,133 unigenes was finally obtained, of which 142,802 (55.54%) unigenes showed significant BLAST results. This is the first transcriptome study of *J. exsertifolia* and provides references for future research of STSs for unique liverworts.

### 4.1. Biosynthetic Genes of Sesquiterpene Backbone for Jungermannia exsertifolia

**Figure 7:**
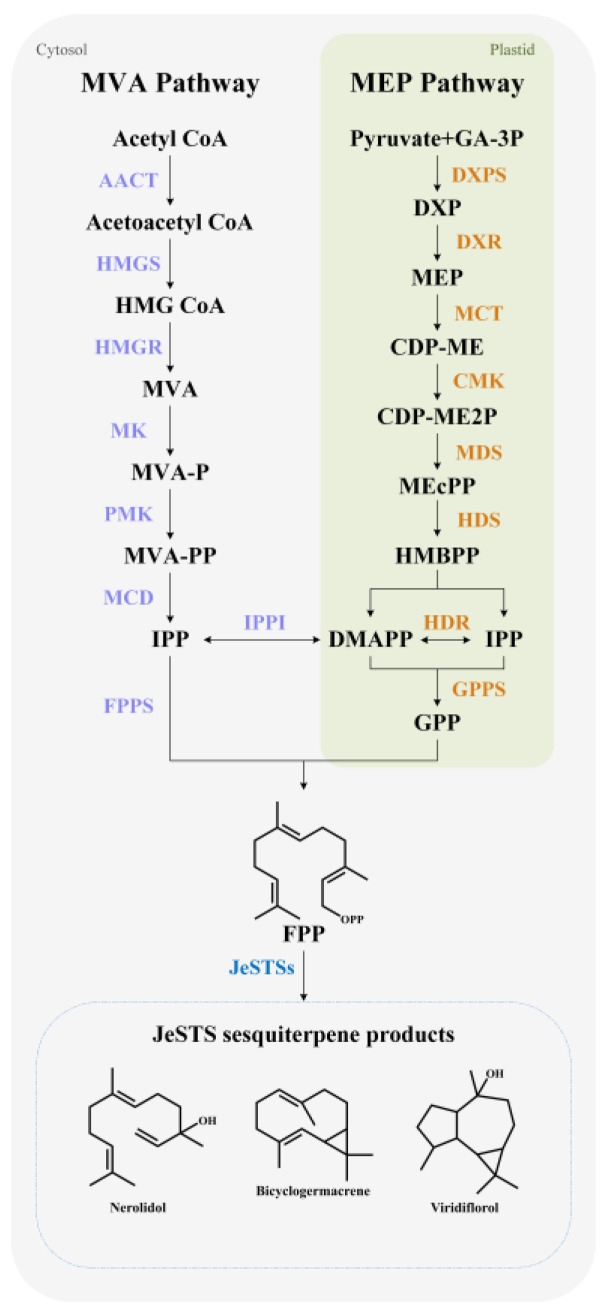
Metabolic pathway for the biosynthesis of sesquiterpenes in *Jungermannia exsertifolia*.

The most basic structural units required for the biosynthesis of *J. exsertifolia* sesquiterpenes are isopentenyl diphosphate (IPP) and its isomer dimethylallyl diphosphate (DMAPP) [34]. The biosynthesis of *J. exsertifolia* sesquiterpenes is mainly through either the MVA pathway or the 2-C-methyl-D-erythritol-4-phosphate (MEP) pathway [35]. The MVA pathway begins with acetyl-CoA while the MEP pathway begins with pyruvate and glyceraldehyde triphosphate. Then, with the catalysis of farnesyl diphosphate synthase, one molecule of IPP and two molecules of DMAPP are connected to form FPP. Subsequently, FPP is catalyzed by JeSTSs to form sesquiterpenes with various structures (Figure 7) [36].

The KEGG annotation results showed that all the enzymes involved in the MEP and MVA pathways have corresponding genes in the transcriptome of *J. exsertifolia* (Table S5). There are 18 genes participating in the MVA pathway, and more genes are related to the synthesis of acetyl-CoA C-acetyltransferase (AACT), hydroxymethylglutaryl-CoA reductase (HMGR), and isopentenyl-diphosphate δ-isomerase (IPPI). At the same time, there are 12 genes involved in the MEP pathway. In addition, six genes participate in the biosynthesis of FPP. These annotated genes provide useful sequence information to understand the metabolic mechanisms of JeSTSs from *J. exsertifolia*, as well as alternative synthetic elements for the biosynthesis of sesquiterpene natural products.

### 4.2. Functional Identification and Heterologous Expression of JeSTSs

The functional identification of terpene synthases is mainly through the functional verification of recombinant proteins in vitro [37,38] and the functional verification in model strains [14,39]. Putative JeSTSs were identified by in vitro reactions of the purified proteins or lysates containing the target proteins with FPP. JeSTS1 and JeSTS2 could catalyze the formation of the linear sesquiterpene nerolidol from FPP. Additionally, JeSTS4 could catalyze the formation of bicyclogemarene from FPP. In *S. cerevisiae*, JeSTS1 and JeSTS2 were identified as nerolidol synthases due to the fact that their fermentation products were predominantly nerolidol. Meanwhile, JeSTS4 was identified as multiple-product STS producing bicyclogermacrene and viridiflorol, which was reported in our previous work [40]. The catalytic products *β*-funebrene, *β*-himachalene, viridiflorol, and nerolidol of these JeSTSs are consistent with the sesquiterpenes detected in the plant essential oil (Table 1) and suggest the specificity of terpene profiles for Jungermanniaceae species. In addition, the aromadendrene-type sesquiterpene product viridiflorol of JeSTS4 has been evaluated as a potential component against insect attacks and fungal infections [41], and a method for the efficient microbial production of viridiflorol has been developed recently [42]. Nerolidol was the mainly catalytic product of JeSTS1 and JeSTS2 and has been used as a food additive in the food industry [43]. Thus, the heterologous expression of these JeSTSs could provide an efficient alternative for the production of these bioactive sesquiterpenes.

### 4.3. Evolutionary Inferences of JeSTSs

JeSTSs are more like MTPSL-STSs. First, the conserved cofactor binding DDXXD motif in plant STSs is atypical in JeSTSs, whose aspartic acid is replaced by glutamine or alanine (Figure 3). Existing studies have shown that many microbial STSs and STSs from other liverworts have atypical DDXXE or DSXXD motifs [14,44,45]. Second, the sequence similarity between JeSTSs and archetypical plant STSs is low, and the total number of amino acids in each identified JeSTSs is significantly less than that of archetypical plant STSs. In addition, the phylogenetic analysis showed that JeSTSs have a closer relationship with bryophyte STSs and some microbial STSs than archetypical plant STSs (Figure 4). Therefore, JeSTSs are considered to belong to MTPSLs, a new branch of plant terpene synthases, which are widely found in non-seed plants like liverworts, hornworts, and lycophytes [11,14,45]. It is speculated that some genes derived from bacteria or fungi were horizontally transferred to *JeSTS* ancestor genes during early evolution [46]. In the future, the structural composition and localization of *JeSTS* genes need to be further studied at the level of genomics.

## 5. Conclusions

Transcriptome analysis of *J. exsertifolia* was conducted using BGISEQ-500 for the first time in order to mine JeSTSs. A total of 257,133 non-redundant genes was obtained, of which 142,802 (55.54%) unigenes produced significant BLAST results.

JeSTS1 and JeSTS2 were identified as nerolidol synthases, and JeSTS4 was identified as an STS that produced bicyclogermacrene and viridiflorol as the dominating products, based on functional identification in vitro and heterologous expression analysis. Phylogenetic analysis showed that the identified JeSTSs had a phylogenetic relationship with MTPSL-STSs.

The identification of JeSTSs suggests specificity of terpene profiles for Jungermanniaceae species and could provide an efficient alternative to the microbial synthesis of these bioactive sesquiterpenes.

## Figures and Tables

**Table 1:** Sesquiterpenes of essential oils from *Jungermannia exsertifolia*.

NO	Constituent	Retention Time (min)	RI ^a^	Area (%)
1	*β*-Funebrene	14.09	1418	10.3
2	Caryophyllene	14.23	1422	0.4
3	Aromadendrene	14.69	1465	23.5
4	Valencene	14.95	1491	2.4
5	*β*-Himachalene	15.11	1500	0.6
6	Nerolidol	15.51	1546	19.1
7	Viridiflorol	15.88	1587	21.3
8	Cubenol	16.51	1643	0.1
9	Lanceol	17.61	1760	11.6
	Others (not identified)	-	-	10.7

^a^ RI: relative retention index on HP-5MS column.

**Table 3:** Summary of functional annotations for transcriptome.

Values	Total	NT	NR	KEGG	SwissProt	KOG	GO	InterPro	Intersection	Overall ^a^
Number	257,133	21,012	114,656	88,847	79,655	102,176	78,862	102,466	9671	142,802
Percentage	100%	8.17%	44.59%	34.55%	30.98%	39.74%	30.67%	39.85%	3.76%	55.54%

^a^ Overall: the number of transcripts annotated by at least one database.

## Data Availability

Not applicable.

## Supplementary Materials

The following supporting information can be downloaded at: https://www.mdpi.com/article/10.3390/bioengineering10050569/s1, Figure S1: Length distribution of unigenes; Figure S2: Comparison of the mass spectra of the peaks in Figure 6 with the authentic standard nerolidol mass spectra, the authentic standard viridilorol mass spectra, and the compound mass spectra stored in the NIST17 library; Table S1: Terpene synthases used for phylogenetic analysis; Table S2: Primers used in this study; Table S3: Quality metrics of clean reads; Table S4: *JeSTS* candidates information; Table S5: Transcripts involved in the biosynthesis of sesquiterpenes in *Jungermannia exsertifolia*.
