# Supplementary material for: Transcriptome Analysis and Identification of Sesquiterpene Synthases in Liverwort Jungermannia exsertifolia
Source: Bioengineering (Basel). 2023 May 9;10(5):569. doi: 10.3390/bioengineering10050569 (PMC10215318; doi:10.3390/bioengineering10050569)
Supplement: Supplementary file 1 [file bioengineering-10-00569-s001.zip › bioengineering-2369432-supplementary.pdf]

Supporting Information

Transcriptome Analysis and Identification of Sesquiterpene Synthases in Liverwort *Jungermannia exsertifolia*

Xiaoguang Yan[1,2,3,4,†], Yukun Li[1,2,3,†], Weiguo Li[1,2,3,4], Dongmei Liang[1,2,3,4], Shengxin Nie[1,2,3], Ruiqi Chen[1,2,3], Jianjun Qiao[1,2,3,4], Mingzhang Wen[1,2,3,*] and Qinggele Caiyin[1,2,3,*]

[1] Department of Pharmaceutical Engineering, School of Chemical Engineering and Technology, Tianjin University, Tianjin 300072, China

[2] Key Laboratory of Systems Bioengineering (Ministry of Education), Tianjin University, Tianjin 300072, China

[3]SynBio Research Platform, Collaborative Innovation Center of Chemical Science and Engineering (Tianjin), Tianjin 300072, China

[4] Zhejiang Institute of Tianjin University, Shaoxing 312300, China

[*] Correspondence: wenmz@tju.edu.cn (M.W.); qinggele@tju.edu.cn (Q.C.)

[†] These authors contributed equally to this work.

Table S1. Terpene synthases used for phylogenetic analysis.

Terpene synthases name	Species	Accession numbers
Sav_76	*Streptomyces avermitilis*	BA000030.4
slt18	*Streptomyces lactacystinaeus*	AB981719.1
Mg25	*Magnolia grandiflora*	EU366429
Cop4	*Coprinus cinereus*	A8NU13
AeTps1	*Aralia elata*	LC307167.1
Copu3	*Coniophora puteana*	XM_007767788
VvPNCuCad	*Vitis vinifera*	HM807407.1
Sce6369	*Sorangium cellulosum*	AB983208.1
GFTpsC	*Citrus x paradisi*	CQ813505
TPS2	*Piper nigrum*	KU953958
Ovtps4	*Origanum vulgare*	E2E2N7.1
LdTPS5	*Lippia dulcis*	JQ731633.1
MrTPS4	*Matricaria recutita*	KJ020284.1
ZmTPS7	*Zea mays*	AC217050.4_FG007
epi-Eud	*Streptomyces viridochromogenes*	NZ_GG657757
zmeds	*Zea mays*	Q93X23
ZSS2	*Zingiber zerumbet*	AB263738
5I1U	*Streptomyces citricolor*	5I1U_A
3G4D	*Gossypium arboreum*	3G4D_A
HcS	*Kitasatospora*	WP_014133196.1
Agg-lim1	*Abies grandis*	O22340
β-phellandrene synthase	*Abies grandis*	AAF61453
linalool synthase	*Actinidia arguta*	ADD81294
nerolidol synthase 1	*Actinidia chinensis*	AER36088
α-farnesene synthase	*Actinidia deliciosa*	ACO40485
germacrene D synthase	*Actinidia deliciosa*	AAX16121
nerolidol/linalool 1 synthase	*Antirrhinum majus*	ABR24417
myrcene synthase 2	*Antirrhinum majus*	AAO41727
CtGES	*Cinnamomum tenuipilum*	Q8GUE4
d-limonene synthase	*Citrus unshiu*	BAD27257
linalool synthase 2	*Clarkia breweri*	AAD19840
delta-cadinene synthase	*Gossypium arboreum*	Q43714
geraniol synthase	*Ocimum basilicum*	AAR11765
E,E-alpha-farnesene synthase	*Picea abies*	AAS47697

3-carene synthase	*Picea sitchensis*	ADU85928
alpha-farnesene synthase	*Pinus taeda*	Q84KL5
myrcene synthase	*Quercus ilex*	Q93X23
exo-alpha-bergamotene synthase	*Santalum album*	E3W202
SaMonoTPS1	*Santalum album*	B5A434
santalene synthase	*Santalum austrocaledonicum*	E3W203
beta-bisabolene synthase	*Santalum austrocaledonicum*	ADO87003
SspiBS	*Santalum spicatum*	E3W206
santalene/bergamotene synthase	*Solanum habrochaites*	ACJ38409
(-)-alpha-terpineol synthase	*Vitis vinifera*	AAS79352
nerolidol/linalool synthase	*Vitis vinifera*	ADR74212
Mg17	*Magnolia grandiflora*	B3TPQ7.1
cadinol synthase	*Lavandula angustifolia*	AGL98418.1
sesquiterpene synthase 4	*Matricaria chamomilla*	AIG92848.1
ent-kaurene synthase	*Jungermannia subulata*	BAJ39816.1
ent-kaurene synthase2	*Physcomitrella patens*	BAF61135.1
OsCPS	*Oryza satiza*	Q6ET36.1
AaMTPSL1	*Anthoceros agrestis*	MF417641
AaMTPSL3	*Anthoceros agrestis*	MF417642
AaMTPSL4	*Anthoceros agrestis*	MF417643
AaMTPSL6	*Anthoceros agrestis*	MF417645
AaMTPSL7	*Anthoceros agrestis*	MF417646
ApMTPSL1	*Anthoceros punctatus*	MF417637
ApMTPSL2	*Anthoceros punctatus*	MF417647
ApMTPSL3	*Anthoceros punctatus*	MF417638
ApMTPSL4	*Anthoceros punctatus*	MF417639
ApMTPSL6	*Anthoceros punctatus*	MF417636
MpMTPSL3	*Marchantia polymorpha*	APP91788
MpMTPSL4	*Marchantia polymorpha*	APP91789
MpMTPSL5	*Marchantia polymorpha*	APP91790
MpMTPSL7	*Marchantia polymorpha*	APP91792
MpMTPSL9	*Marchantia polymorpha*	APP91797
Liv-IRBN-MTPSL4	*Pityrogramma trifoliata*	APB88774.1
Liv-IRBN-MTPSL2	*Pityrogramma trifoliata*	APB88773.1
Mon-GSXD-MTPSL3	*Myriopteris eatonii*	APB88779.1
Mos-VBMM-MTPSL3	*Anomodon rostratus*	APB88777.1
Mos-QKQO-MTPSL3	*Pseudotaxiphyllum elegans*	APB88776.1

Mon-YJJY-MTPSL1	*Woodsia scopulina*	APB88781.1
Mon-UJTT-MTPSL4	*Pityrogramma trifoliata*	APB88780.1
Hon-ApMTPSL7	*Anthoceros punctatus*	APB88778.1
JeSTS1	*Jungermannia exsertifolia*	MT277446
JeSTS2	*Jungermannia exsertifolia*	MT277447
JeSTS3	*Jungermannia exsertifolia*	MT277448
JeSTS4	*Jungermannia exsertifolia*	MT277449
JeSTS5	*Jungermannia exsertifolia*	MT277450
JeSTS6	*Jungermannia exsertifolia*	MT277451

Table S2. Primers used in this study.

Primer name	Usage	Primer sequences
JeSTS1_TF	subclone *JeSTS1* into the pET28a	CATGCCATGGGTATGGCGGCGTATATCACGC
JeSTS1_TR	subclone *JeSTS1* into the pET28a	CCGCTCGAGAACCACCACCACGA
JeSTS2_TF	subclone *JeSTS2* into the pET28a	CATGCCATGGGTATGGATACCGAAGGCGGC
JeSTS2_TR	subclone *JeSTS2* into the pET28a	CCGCTCGAGGGTCCAATACACACCGG
JeSTS3_TF	subclone *JeSTS3* into the pET28a	CATGCCATGGGTATGACGATTGTTTTACGGCG
JeSTS3_TR	subclone *JeSTS3* into the pET28a	CCGCTCGAGCGTGCTCTGGAAATAGCGG
JeSTS4_TF	subclone *JeSTS4* into the pET28a	CATGCCATGGGTATGGCCACCGAACTCGC
JeSTS4_TR	subclone *JeSTS4* into the pET28a	CCGCTCGAGCTGGTAGCGAACGCTGT
JeSTS5_TF	subclone *JeSTS5* into the pET28a	CATGCCATGGGTATGGCTAGTCCGGCGACC
JeSTS5_TR	subclone *JeSTS5* into the pET28a	CCGCTCGAGGCTCAGCAGAACGGCT
JeSTS6_TF	subclone *JeSTS6* into the pET28a	CATGCCATGGGTATGGCGAGCCCACTGC
JeSTS6_TR	subclone *JeSTS6* into the pET28a	CCGCTCGAGCAGCTGAACCGTAACGCCG
JeSTS1_SF	subclone *JeSTS1* into the pESC-LEU	CGGGATCCATGGCGGCGTATATCACGC
JeSTS1_SR	subclone *JeSTS1* into the pESC-LEU	CCCAAGCTTTCAAACCACCACCACGA
JeSTS2_SF	subclone *JeSTS2* into the pESC-LEU	CGGGATCCATGGATACCGAAGGCGGC
JeSTS2_SR	subclone *JeSTS2* into the pESC-LEU	CCCAAGCTTTCAGGTCCAATACACACCGG

JeSTS3_SF	subclone *JeSTS3* into the pESC-LEU	CGGGATCCATGACGATTGTTTTTACGGCG
JeSTS3_SR	subclone *JeSTS3* into the pESC-LEU	CCCAAGCTTTCACGTGCTCTGGAAATAGCGG
JeSTS4_SF	subclone *JeSTS4* into the pESC-LEU	CGGGATCCATGGCCACCGAACTCGC
JeSTS4_SR	subclone *JeSTS4* into the pESC-LEU	CCCAAGCTTTCACTGGTAGCGAACGCTGT
JeSTS5_SF	subclone *JeSTS5* into the pESC-LEU	CGGGATCCATGGCTAGTCCGGCGACC
JeSTS5_SR	subclone *JeSTS5* into the pESC-LEU	CCCAAGCTTTCAGCTCAGCAGAACGGCT
JeSTS6_SF	subclone *JeSTS6* into the pESC-LEU	CGGGATCCATGGCGAGCCCACTGC
JeSTS6_SR	subclone *JeSTS6* into the pESC-LEU	CCCAAGCTTTCACAGCTGAACCGTAACGCCG

Table S3. Quality metrics of clean reads.

Sample	Total raw reads (M)	Total clean reads (M)	Total clean bases (Gb)	Clean reads Q20 (%)[a]	Clean reads Q30 (%)[b]	Clean reads ratio (%)
J1	114.54	110.35	11.04	95.61	87.10	96.34
J2	114.54	110.57	11.06	96.21	88.51	96.53

[a] Clean Reads Q20 (%): The rate of bases in which quality is greater than value 20 in clean reads; [b] Clean Reads Q30 (%): The rate of bases in which quality is greater than value 30 in clean reads.

Table S4. *JeSTS* candidate information.

Gene name	Transcript ID	Num of bases (bp)	Num of amino acids	Predicted molecular weight (kDa)
JeSTS1	CL7806.Contig2_All	1,194	397	46.2
JeSTS2	Unigene11740_All	1,062	353	40.2
JeSTS3	Unigene14583_All	1,353	450	51.5
JeSTS4	Unigene16378_All	1,140	379	43.8
JeSTS5	Unigene3358_All	1,696	564	63.1
JeSTS6	Unigene35759_All	1,056	351	39.6

Table S5. Transcripts involved in the biosynthesis of sesquiterpenes in *Jungermannia exsertifolia.*

Enzyme name	Abbreviation	EC number	Number of unigenes with ORF
Acetyl-CoA C-acetyltransferase	AACT	EC 2.3.1.9	5
Hydroxymethylglutaryl-CoA synthase	HMGS	EC 2.3.3.10	2
Hydroxymethylglutaryl-CoA reductase	HMGR	EC 1.1.1.34	3
Mevalonate kinase	MK	EC 2.7.1.36	2
Phosphomevalonate kinase	PMK	EC 2.7.4.2	2
Diphosphomevalonate decarboxylase	MCD	EC:4.1.1.33	1
1-Deoxy-D-xylulose-5-phosphate synthase	DXPS	EC 2.2.1.7	1
1-Deoxy-D-xylulose-5-phosphate reductoisomerase	DXR	EC 1.1.1.267	2
4-Diphosphocytidyl-2-C-methyl-D-erythritol synthase	MCT	EC 2.7.7.60	2
4-Diphosphocytidyl-2-C-methyl-D-erythritol kinase	CMK	EC 2.7.1.148	1
2-C-methyl-D-erythritol 2,4-cyclodiphosphate synthase	MDS	EC 4.6.1.12	2
4-Hydroxy-3-methylbut-2-enyl diphosphate synthase	HDS	EC 1.17.7.1	2
1-Hydroxy-2-methyl-butenyl 4-diphosphate reductase	HDR	EC 1.17.7.4	2
Isopentenyl-diphosphate δ-isomerase	IPPI	EC 5.3.3.2	3
Farnesyl diphosphate synthase	FPPS	EC 2.5.1.10	6

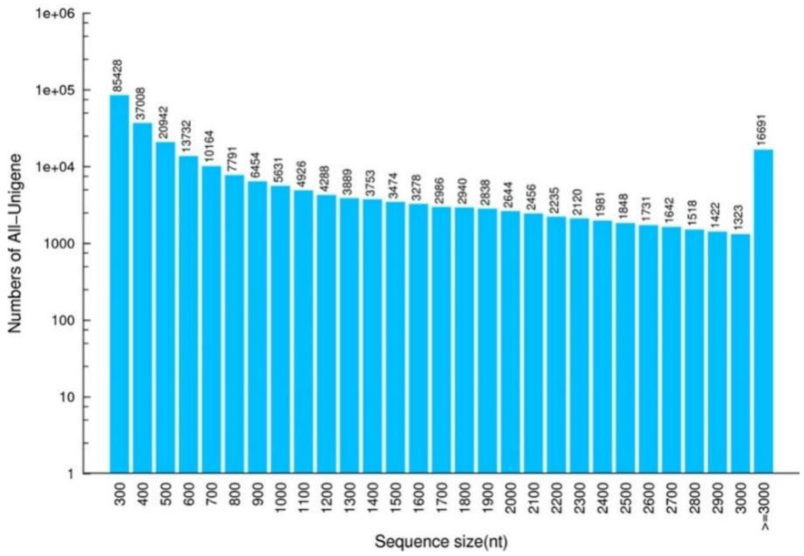

Figure S1. Length distribution of unigenes.

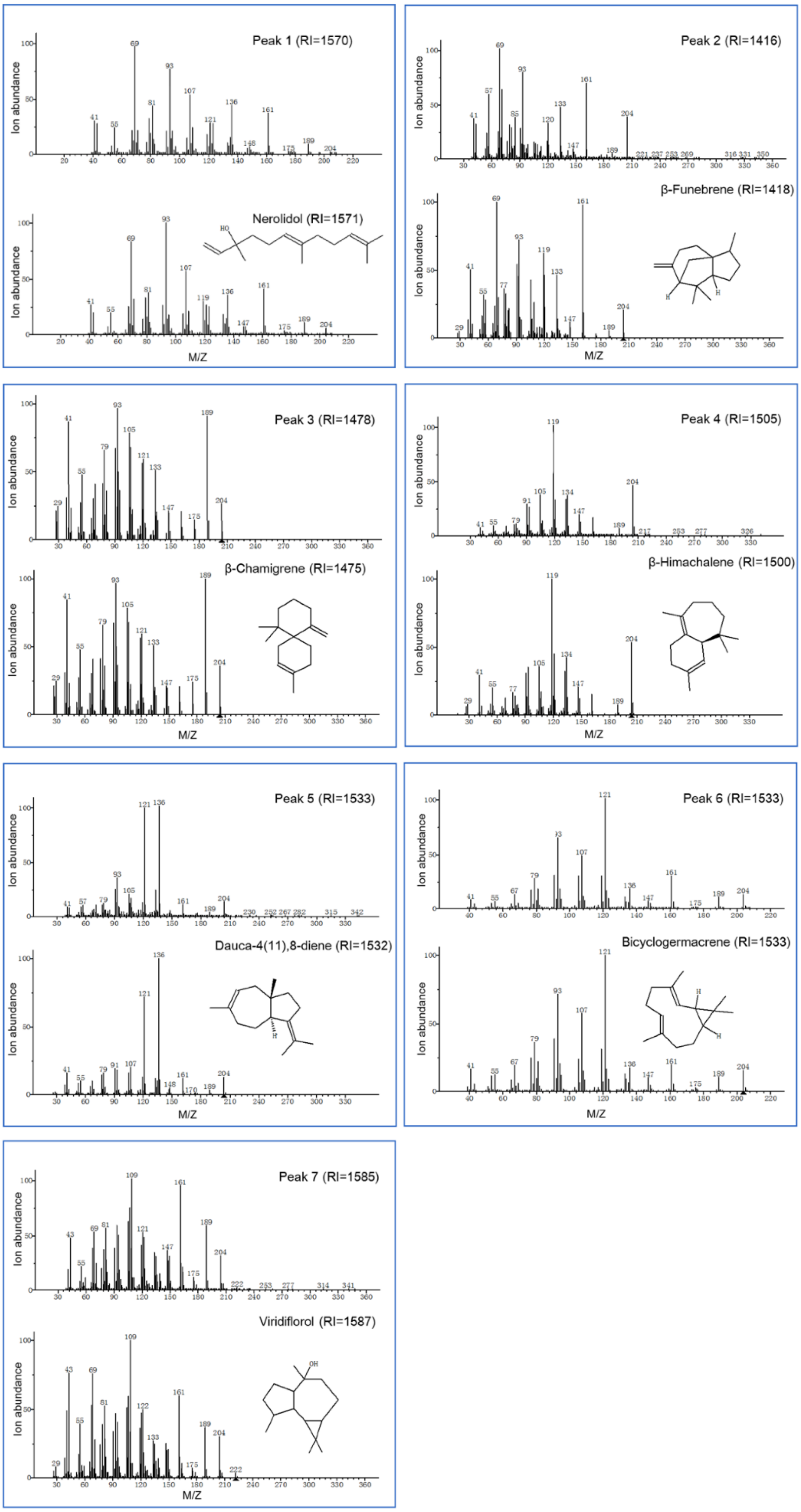

Peak 1 (RI=1570)
Nerolidol (RI=1571)
Peak 2 (RI=1416)
β-Funebrene (RI=1418)
Peak 3 (RI=1478)
β-Chamigrene (RI=1475)
Peak 4 (RI=1505)
β-Himachalene (RI=1500)
Peak 5 (RI=1533)
Dauca-4(11),8-diene (RI=1532)
Peak 6 (RI=1533)
Bicyclogermacrene (RI=1533)
Peak 7 (RI=1585)
Viridiflorol (RI=1587)
Ion abundance
M/Z

Figure S2. Comparison of the mass spectra of the peaks in Figure 6 with the authentic standard nerolidol mass spectra, the authentic standard viridilorol mass spectra, and the compound mass spectra stored in the NIST17 library.
